# *Schisandra* polysaccharide inhibits hepatic lipid accumulation by downregulating expression of SREBPs in NAFLD mice

**DOI:** 10.1186/s12944-016-0358-5

**Published:** 2016-11-16

**Authors:** Chun-Mei Wang, Rong-Shuang Yuan, Wen-Yue Zhuang, Jing-Hui Sun, Jin-Ying Wu, He Li, Jian-Guang Chen

**Affiliations:** 1 Department of Pharmacology, College of Pharmacy, Beihua University, No. 3999 Binjiang East Road, Fengman District, Jilin City, Jilin Province People’s Republic of China; 2 Department of Molecular Biology, College of Laboratory Medicine, Beihua University, Jilin, Jilin 132013 China

**Keywords:** *Schisandra chinensis* (*Schisandra*), Polysaccharide, Nonalcoholic fatty liver disease (NAFLD), Sterol regulatory element-binding proteins (SREBPs)

## Abstract

**Background:**

Hepatoprotective effects of Chinese herbal medicine *Schisandra Chinensis* (*Schisandra*) have been widely investigated. However, most studies were focused on its lignan extracts. We investigated the effects of *Schisandra* polysaccharide (SCP) in a mouse model of non-alcoholic fatty liver disease (NAFLD), and studied its effect on sterol regulatory element binding proteins (SREBPs) and the related genes.

**Methods:**

The mouse model of NAFLD was established by feeding mice with a high-fat diet for 16 weeks. Effect of SCP-treatment (100 mg/kg, once daily for 12 weeks) on biochemical parameters and liver histopathology was assessed. Relative levels of sterol regulatory element-binding proteins (SREBPs) and their gene expressions were determined by quantitative real-time polymerase chain reaction and Western Blot.

**Results:**

SCP significantly reduced the liver index by 12.0%. Serum levels of triglycerides (TG), total cholesterol (TC), low-density lipoprotein cholesterol, alanine aminotransferase and aspartate aminotransferase were decreased by 31.3, 28.3, 42.8, 20.1 and 15.5%, respectively. Serum high-density lipoprotein cholesterol was increased by 26.9%. Further, SCP lowered hepatic TC and TG content by 27.0% and 28.3%, respectively, and alleviated fatty degeneration and necrosis of liver cells. A significant downregulation of mRNA and protein expressions of hepatic lipogenesis genes, SREBP-1c, fatty acid synthase and acetyl-CoA carboxylase, and the mRNA expression of liver X receptor α (LXRα) was observed in NAFLD mice treated with SCP. SCP also significantly reduced the hepatic expression of SREBP-2 and 3-hydroxy-3-methylglutaryl coenzyme A reductase (HMGCR).

**Conclusion:**

These findings demonstrate the hepatoprotective effects of SCP in a mouse model of NAFLD; the effects may be mediated via downregulation of LXRα/SREBP-1c/FAS/ACC and SREBP-2/HMGCR signaling pathways in the liver.

## Background

Non-alcoholic fatty liver disease (NAFLD) is one of the most common precursors of chronic liver diseases (such as hepatic fibrosis and liver cancer), and metabolic disorders such as obesity, type 2 diabetes, and atherosclerosis [1, 2]. In recent years, the incidence of NAFLD has dramatically increased, with an approximate incidence of about 20% in general population, and > 60% in patients with obesity and type 2 diabetes [3]. Though the pathogenesis of NAFLD is not completely understood, disordered lipid metabolism is considered a key factor. Apart from interventions such as diet and exercise, no pharmaceutical agents are currently available for the treatment of NAFLD. Therefore, studies on key factors that regulate lipid metabolism in the liver, with the aim to discover novel drug targets for the prevention and treatment of NAFLD are being carried out.

Sterol regulatory element binding proteins (SREBPs) are transcription factors that regulate the biosynthesis of cholesterol, fatty acids, triglycerides, and the differentiation of adipocytes. Abnormal expression of SREBPs may have a role in the pathogenesis of NAFLD. Indeed, SREBPs are potential therapeutic targets for NAFLD [4, 5].

*Schisandra* is a traditional Chinese herbal remedy for chemical- and virus-induced liver injury. Aqueous and ethanolic extracts of *Schisandra* were recently shown to have a significant lipid-lowering effect in mice models of fatty liver [6]. Its active components and synthetic derivatives such as, bifendate and bicyclol, also showed a similar effect [7, 8]. The effect may be mediated via downregulation of SREBP-1 expression. Moreover, anti-oxidative, hypoglycemic and anti-inflammatory effects of *Schisandra* have also been demonstrated [9]. Based on these findings, a potential role of *Schisandra* in the treatment of NAFLD is of considerable interest. The active ingredients of *Schisandra* include lignans and polysaccharides [10]; most studies till date have focused on the ethanol extracts and lignans of *Schisandra*.

Pan et al. [11] reported that *Schisandra* B, the most abundant and active dibenzocyclooctadiene derivative isolated from the fruit of *Schisandra chinensis*, increased serum and hepatic triglyceride levels in mice at a dose of 1 g/kg. Moreover, it was used to develop an experimental model of acute hypertriglycerdemia, which indicates that lignans are not ideal for the treatment of NAFLD.

Plant polysaccharides have recently evoked considerable interest for their lipid-lowering, hypoglycemic, and immunomodulatory properties. Our laboratory has been involved in research on SCP since 2010 and our previous studies revealed a hypolipidemic, anti-obesity and hepatoprotective effect of SCP against chemical-induced liver injury in obese mice. The findings suggest SCP to be the main component of *Schisandra* responsible for its lipid-lowering and hepatoprotective effects.

In this study, a NAFLD mouse model was established by feeding mice with a high-fat diet. Effects of *Schisandra* polysaccharide on NAFLD were investigated, including its effect on SREBPs and the related genes.

## Methods

### Reagents

*Schisandra chinensis* was purchased from Jilin Province, Jian. *Schisandra* seedlings base was identified by Professor Li-Hua Zhang at the College of Pharmacy, Beihua University according to the identification standard of the Pharmacopoeia of the People’s Republic of China (2010 Edition). Kits for measurement of serum levels of TG, TC, LDL-C, HDL-C, ALT, and AST were purchased from BioSino Biotechnology & Science Inc. (Beijing, China). Kits for measuring tissue TC and TG were purchased from Applygen Technologies Inc. (Beijing, China). Primers were synthesized by Dingguo Changsheng Biotechnology Co. Ltd. (Beijing, China). Polyclonal antibodies to SREBP-1c, SREBP-2, HMGCR, LXRα, ACC, and FAS were purchased from Abcam (London, England). Reagents for Western Blot and RT-PCR were purchased from Sigma Aldrich.

### Extraction and characterization of SCP

#### Extraction of SCP

SCP was prepared at the Jilin Province *Schisandra* Industrialization and Engineering Research Center. Briefly, lipid-soluble substances were removed by supercritical CO_2_ extraction; the single factor test of SCP extraction was used to determine the ratio of material to water (1:20, 1:30, and 1:40), extraction temperature (80 °C, 90 °C, and 100 °C) and extraction time (2, 3, and 4 h); orthogonal test (L_9_3^3^) were used to determine the optimal conditions (the ratio of material to water: 1:30; extraction temperature: 100 °C, and extraction time: 3 h). The concentrated extract was precipitated in about 5× volume of 95% ethanol for 6 h, which was then centrifuged and dried to obtain crude polysaccharides of *Schisandra*. The crude polysaccharides were deproteinized by the enzymolysis method combined with trichloroacetic acid-n-butanol method, namely, enzyme 2%, pH 5.0, and enzymatic hydrolysis at 60 °C for 2 h, and then treated with an equal volume of trichloroacetic acid-n-butanol solution for 5 times. Activated carbon was used for decolorization. The optimum conditions were: 60 °C, pH 4.0, 1.5% activated carbon, stirring for 50 min, and 91.7% decolorization rate. The refined SCP obtained by the above methods was used in animal experiments.

### Characterization of SCP

Using D-glucose as the reference, the total sugar content was determined by phenol-sulfuric acid method. Using galacturonic acid as the reference, the uronic acid content was measured with meta-hydroxydiphenyl method. The protein content was detected with Coomassie brilliant blue method.

### Animal experimentation

Male C57BL/6 mice, weighing 19–21 g, were provided by Beijing Vital River Laboratory Animal Technology Co. Ltd. (Beijing, China) [license number: SCXK (Beijing) 2012–0001, SPF]. The mice were raised in separate cages maintained at 18–22 °C (relative humidity 40–60%), under normal lighting conditions (lights on from 7 a.m. to 7 p.m.), and with ad libitum access to water. The normal (AIN-93) and high-fat diet (AIN-93 modified) for experimental mice was purchased from Changchun City Yisi Laboratory Animal Technology Co. Ltd (Changchun, Jilin, China) (Table 1). All procedures were approved by the Ethics Committee for Use of Experimental Animals at the Beihua University (Jilin, China).

**Table 1:** Composition of experimental diet ND and HFD

Ingredients	ND	HFD
Corn Starch (%)	72.07	59.57
Casein (%)	14.0	14.0
Soybean Oil (%)	4.0	4.0
Lard (%)	-	10.0
Sucrose (%)	5.0	5.0
Cholesterol (%)	-	2.0
Sodium Taurocholate (%)	-	0.5
Mixture of Vitamins (%)	1.0	1.0
Mixture of Mineral (%)	3.5	3.5
L-cystine (%)	0.18	0.18
Choline Bitartrate (%)	0.25	0.25
Hydroquinone (g/kg)	0.008	0.008
Energe (Kal/kg)	3802	5443

*ND* normal diet, *HFD* high-fat diet

#### Establishment of NAFLD model and drug administration

Twenty out of 40 male mice were randomly divided into a normal control group (CON, *n* = 10) and a normal control plus SCP (100 mg/kg) group (CON + SCP, *n* = 10), and the mice were fed on the normal diet. The remaining 20 mice were used to establish NAFLD model. After being fed on high-fat diet for 4 weeks, the mice were denied food but not water for 12 h. Blood was collected from eyeballs with a capillary glass tube and the serum total cholesterol and triglyceride levels measured to ascertain the establishment of the model.

Subsequently, these mice were randomly divided into NAFLD group (NAFLD) and NAFLD plus SCP (100 mg/kg) group (NAFLD + SCP), *n* = 10 in each group. Mice in the CON and NAFLD groups were administered equal volumes of water and those in the CON + SCP and NAFLD + SCP groups were administered 100 mg/kg SCP orally, once daily for 12 weeks. The dosage of SCP used in this study was based on the pre experiment [12]. Body weights were measured every week. After the last administration of water or SCP, all mice were denied food but not water for 12 h, and anesthetized with ether, then 0.8–1.0 mL of blood was collected from each mouse by removing eyeballs. Serum was separated by centrifugation and stored at −80 °C.

The peritoneal cavity was opened along the abdominal middle line, and the liver carefully isolated and resected. The liver index (wet liver weight/body weight × 100%), overall morphology and the surface gloss of the liver was documented. Hepatic tissue was washed with cold saline and divided into three parts: the first part was fixed with 10% neutral formaldehyde for histopathological examination; the second part was prepared into homogenates for estimation of liver lipids; and the third part was preserved at −80 °C for studying the expression of SREBPs and related genes.

### Measurement of serum biochemical markers

TG, TC, LDL-C, and HDL-C levels in the serum were detected using enzymatic methods as per manufacturer’s instructions (BioSino Biotechnology & Science Inc., Beijing, China).

### Measurement of hepatic lipid content

One hundred milligrams of liver tissue was cut into pieces and placed in tubes containing 10% lysate buffer. After being left to stand for 1h, the sample was homogenized at 16,000 rpm in ice-water (the homogenization process was repeated 3 times; the duration of each homogenization process was 5 s, followed by a 3 s interval). The homogenate was heated at 70 °C in a water bath for 10 min and centrifuged twice at 3500 × g for 5 min and the supernatant obtained. TC and TG content in the supernatant was detected as per manufacturer’s instructions (Applygen Technologies Inc., Beijing, China).

### Histopathological examination

Liver tissue sections were stained with hematoxylin-eosin (H&E). The images were digitized by Nikon Digital Camera attached with BH51 microscope (Olympus, Tokyo, Japan). An experienced histopathologist blinded to the treatment protocol evaluated the stained slides. Each sample was observed at 100× magnification.

### RNA extraction and real-time reverse transcription polymerase chain reaction

Total RNA was extracted from mouse liver tissues using Trizol reagent (Invitrogen, USA) to examine the effects of SCP on the expression of TG- and TC-related genes. Reverse transcription (RT) of total RNA (1 μg) was performed as per manufacturer’s instructions (Promega, Beijing, China) in a final volume of 20 μL. PCR primer sequences were designed using primer 6.0 software and synthesized by Dingguo Changsheng Biotechnology Co. Ltd (Beijing, China). The gene expression levels were analyzed in duplicate using a SYBR Green kit (Promega, Beijing, China), according to the manufacturer’s instructions, on real-time PCR System (ABI 7500, Biometra, Germany). The primer lengths and annealing temperatures are listed in Table 2. The ^∆∆^Ct method was used for relative quantification. The relative gene expression was normalized to GAPDH expression, and the ratios presented as arbitrary units.

**Table 2:** Primers used for quantitative real-time polymerase chain reaction

Genes	Primer sequences	Length
SREBP-1c	Forward 5′-GGAGACATCGCAAACAAGC-3′	273bp
	Reverse 5′-GGTAGACAACAGCCGCATC-3′	
SREBP-2	Forward 5′-CACCCATACTCAGGCTCG-3′	133bp
	Reverse 5′-GCTTCACAAAGACGCTCAA-3′	
FAS	Forward 5′-ATCGCCTATGGTTGTTG-3′	127bp
	Reverse 5′-TCACGACTGGAGGTTCTA-3′	
ACC	Forward 5′-TATCCCAACTCTTCCCTG-3′	116bp
	Reverse 5′-CCTTCACATAGCCTTTCTC-3′	
HMGCR	Forward 5′-CTTGACGCTCTTGTGGA-3′	259bp
	Reverse 5′-CCCTTTGGGTTACGG-3′	
LDLR	Forward 5′-GGAGGTGACCAACAATAGAA-3′	190bp
	Reverse 5′-TACGCTGCCTGGGACTG-3′	
LXR	Forward 5′-CTCAATGCCTGATGTTTCTCC-3′	154bp
	Reverse 5′-TGACTCCAACCCTATCCCTAA-3′	

### Western blot analysis

Protein samples were prepared from liver tissues by homogenization with ice-cold lysate buffer for 1 h, and the tissue lysate samples centrifuged at 15,000 × g for 20 min at 4 °C. The supernatants were collected and the protein concentrations measured using Bradford assay (Bio-rad protein assay kit). For detection of SREBP-1c, SREBP-2, FAS, ACC, HMGCR and LXRα protein expressions, protein samples (60–80 μg) were denatured by boiling for 5 min, separated by 10% SDS-polyacrylamide gel, electroblotted at 4 °C and transferred onto polyvinylirdene diflouride (PVDF) membranes (Millipore, USA). The membranes were blocked in 5% non-fat milk for 2 h at room temperature and then incubated with rabbit polyclonal antibodies against SREBP-1c, SREBP-2, FAS, ACC, HMGCR and LXRα (diluted 1:1,000) with gentle agitation overnight at 4 °C. The membranes were washed 3 times for 10 min each with 15 mL of TBST and then incubated with second antibody (1:1000 goat Anti-rabbit IgG Horseradish Peroxidase Conjugate (Santacruz Biotechnology) at room temperature for 2 h. The protein was then visualized using an enhanced chemiluminescence solution and Kodak X-ray film. An imaging densitometer was used to scan the protein bands; quantification was performed using the Image Analysis Software.

### Statistical analysis

All data are expressed as mean ± Standard Deviation. “*n*” denotes the sample size in each group. Between-group differences were assessed using one-way Analysis of Variance (ANOVA) with post hoc test for multiple comparisons. SPSS software (version 20.0 for Windows) was used for statistical analysis. *P* < 0.05 was considered to be statistically significant.

## Results

### Characterization of SCP

SCP was in a milky white powder form and its yield was 10.62% (the percentage of SCP to the dry weight of raw medicinal material). The total sugar content, uronic acid content and protein content of SCP were 68.2%, 2.96%, and 0.71 mg/g, respectively.

### Effects of SCP on the liver weight and liver index

At 12 weeks, liver weights in the NAFLD group were increased by 30.5% as compared to that in the control mice (*P* < 0.05). However, no significant difference was observed with respect to body weight, which may reflect the effect of sodium taurocholate on the appetite of mice [13]. The liver weight and liver index were reduced by 15.3% and 12.0%, respectively, in NAFLD + SCP group when compared with NAFLD group (*P* < 0.05) (Table 3).

**Table 3:** Effects of SCP on body weight, liver weight and liver index in NAFLD mice (*n =* 10)

Group	Body weight (g)	Liver weight (g)	liver weight/body weight ratio
CON	29.57 ± 2.25	1.05 ± 0.18	3.52 ± 0.85
CON + SCP	28.94 ± 1.84	1.02 ± 0.14	3.47 ± 0.68
NAFLD	30.52 ± 2.34	1.37 ± 0.11^*^	4.58 ± 0.60^*^
NAFLD + SCP	28.56 ± 1.97	1.16 ± 0.19^#^	4.03 ± 0.71^#^

CON: treated with saline 0.1 mL/10g; NAFLD: treated with saline 0.1 mL/10g; CON + SCP and NAFLD + SCP: treated with SCP (100 mg/kg). Data are expressed as mean ± SD (*n* = 10) and analyzed using one-way ANOVA with post hoc test

*SCP Schisandra polysaccharide, NAFLD non-alcoholic fatty liver disease, SD* standard deviation*, ANOVA,* analysis of variance

^*^ *P* < 0.05 *vs.* CON and CON + SCP groups; ^#^ *P* < 0.05 *vs.* model group

### Effects of SCP on blood lipid levels

As shown in Table 4, compared with those in CON group, serum TC, TG and LDL-C levels in the NAFLD group were increased by 100.3%, 72.9%, and 138.9%, respectively (*P* < 0.01 or *P* < 0.05), while serum HDL-C levels were decreased by 32.9% (*P* < 0.05). As compared to that in NAFLD group, the serum TC, TG and LDL-C levels in NAFLD + SCP group were decreased by 28.3%, 31.3% and 42.8%, respectively (*P* < 0.05), while serum HDL-C levels were increased by 26.9% (*P* < 0.05), which indicates alleviation of dyslipidemia in NAFLD mice by SCP. No effect of SCP on blood lipid levels was observed in control mice.

**Table 4:** Effects of SCP on blood lipids in NAFLD mice (*n* = 10)

Group	TC (mmol/L)	TG (mmol/L)	LDL-C (mmol/L)	HDL-C (mmol/L)
CON	2.89 ± 0.26	0.85 ± 0.11	0.90 ± 0.05	1.58 ± 0.54
CON + SCP	2.85 ± 0.45	0.88 ± 0.25	0.84 ± 0.08	1.53 ± 0.32
NAFLD	5.79 ± 0.70^**^	1. 47 ± 0.31^*^	2.15 ± 0.61^**^	1.06 ± 0.21^*^
NAFLD + SCP	4.15 ± 0.86^#^	1.01 ± 0.21^#^	1.23 ± 0.24^#^	1.45 ± 0.38^#^

CON: treated with saline 0.1 mL/10g; NAFLD: treated with saline 0.1 mL/10g; CON + SCP and NAFLD + SCP: treated with SCP (100 mg/kg). Data are expressed as mean ± Standard deviation (*n* = 10) and analyzed using one-way ANOVA with post hoc test. ^*^ *P* < 0.05, ^**^ *P* < 0.01 *vs.* CON and CON + SCP group; ^#^ *P* < 0.05,^##^ *P* < 0.01 *vs.* model group

*SCP Schisandra polysaccharide, NAFLD* non-alcoholic fatty liver disease*, TG* triglycerides*, TC,* total cholesterol*, LDL-C,* low-density lipoprotein cholesterol*, SD* standard deviation*, ANOVA,* analysis of variance

### Effects of SCP on the accumulation of hepatic lipids

**Fig. 1:**
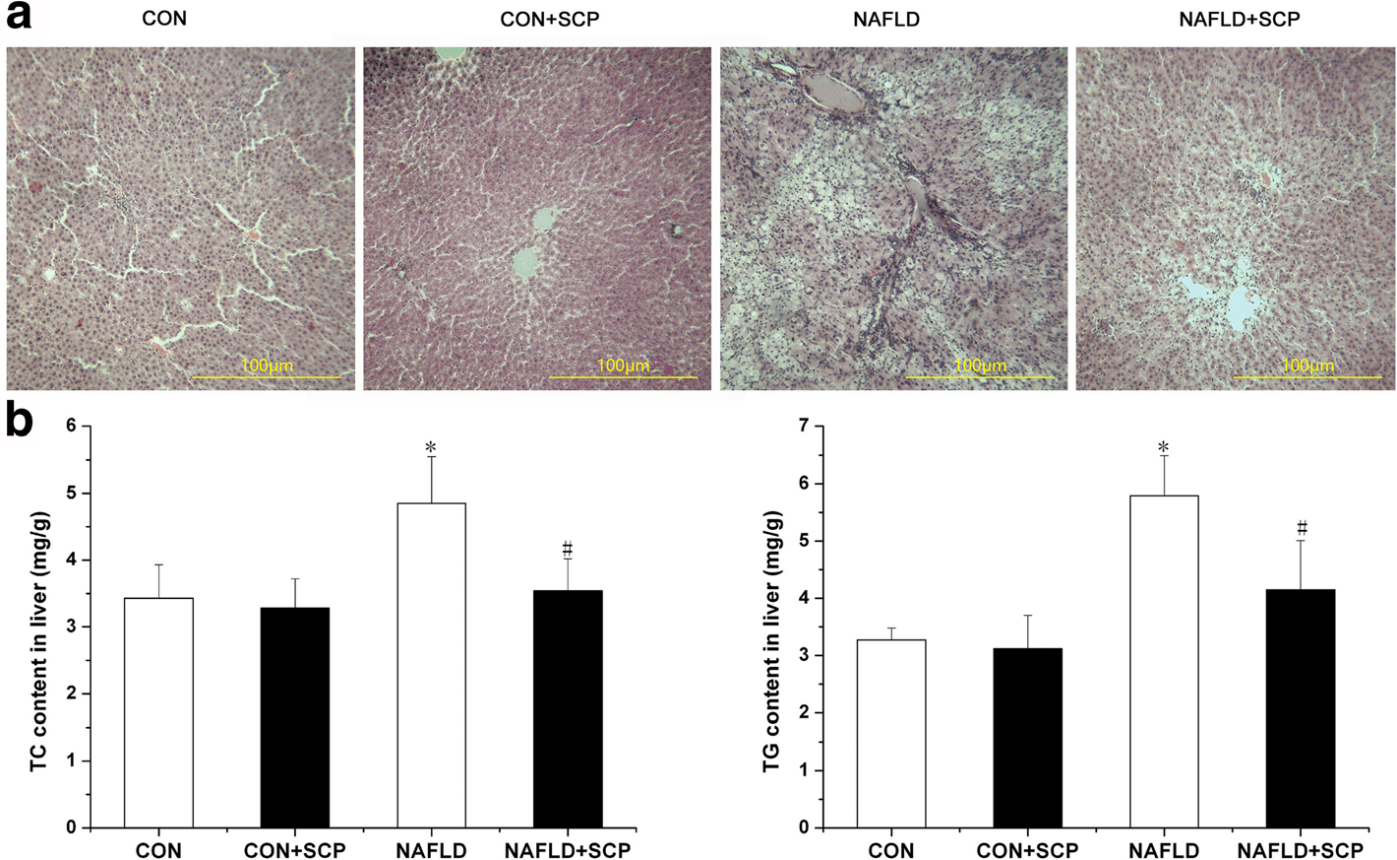
Effects of SCP on the pathomorphology of liver and hepatic lipid content in NAFLD mice. **a** H&E stained sections of liver tissue (×100); **b** Quantitative analysis of hepatic total cholesterol (TC) and triglyceride (TG) content. Mice in SCP group were administered SCP at 100 mg/kg/day for 12 weeks. Data are expressed as means ± SD (*n* = 10). Between-group differences were assessed using one-way ANOVA with post hoc test. ^*^ *P* < 0.05 *vs.* CON and CON + SCP groups; ^#^ *P* < 0.05 *vs.* model group. *SCP, Schisandra polysaccharide; NAFLD, non-alcoholic fatty liver disease; H&E, hematoxylin and eosin; SD, standard deviation; ANOVA, analysis of variance*

Histopathological examination of liver tissues showed a normal intact structure of hepatic lobules, round shape of liver cells, central location of nuclei, pink dyeing of the cytoplasm, and normal structure of portal area around hepatic lobules in the CON group (Fig. 1a-CON). In the NAFLD group, the structural disorder of hepatic lobules, dilation of portal area-central vein, fatty degeneration of liver cells within hepatic lobules, mixed fatty degeneration characterized by macrovesicular steatosis, enlargement of liver cell volume, visible cavitation within the cytoplasm, and hepatocellular spotty necrosis within hepatic lobules was noted (Fig. 1a-NAFLD). Compared with NAFLD group, a relatively normal structure of hepatic lobules, significantly improved fatty degeneration of liver cells in hepatic lobules and less visible necrosis of hepatocytes was noted in NAFLD + SCP group (Fig. 1a-NAFLD+ SCP). The TC and TG content in liver tissue of mice in NAFLD group were significantly increased by 41.4% and 77.1%, respectively (*P* < 0.05) when compared with those in CON group. While TC and TG content of liver tissue in NAFLD + SCP group was significantly reduced by 27.0% and 28.3%, respectively, when compared to NAFLD group (*P* < 0.05), which indicates that SCP could improve lipid accumulation in the liver tissue and protect the liver cells (Fig. 1b).

### Effects of SCP on serum aminotransferase levels

**Fig. 2:**
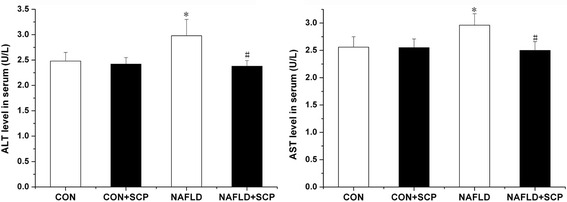
Effects of SCP on serum aminotransferases in NAFLD mice. Mice in SCP group were administered SCP (100 mg/kg/day) for 12 weeks. Data are expressed as means ± SD (*n* = 10); Between-group differences were assessed using one-way ANOVA with post hoc test. ^*^ *P* < 0.05 *vs.* CON and CON + SCP groups; ^#^ *P* < 0.05 *vs.* model group. *SCP, Schisandra polysaccharide; NAFLD, non-alcoholic fatty liver disease; SD, standard deviation; ANOVA, analysis of variance*

ALT and AST levels are considered as “gold standard” markers of hepatic cell injury. The results showed that compared with those in CON group, serum ALT and AST levels of mice were elevated by 20.2 and 15.6%, respectively (*P* < 0.05), in the NAFLD group, while the levels were reduced by 20.1 and 15.5%, respectively (*P* < 0.05), in the NAFLD + SCP group as compared to those in the NAFLD group. These findings suggest a hepatoprotective effect of SCP (Fig. 2).

### Effects of SCP on mRNA and protein expressions of genes coding for SREBPs

**Fig. 3:**
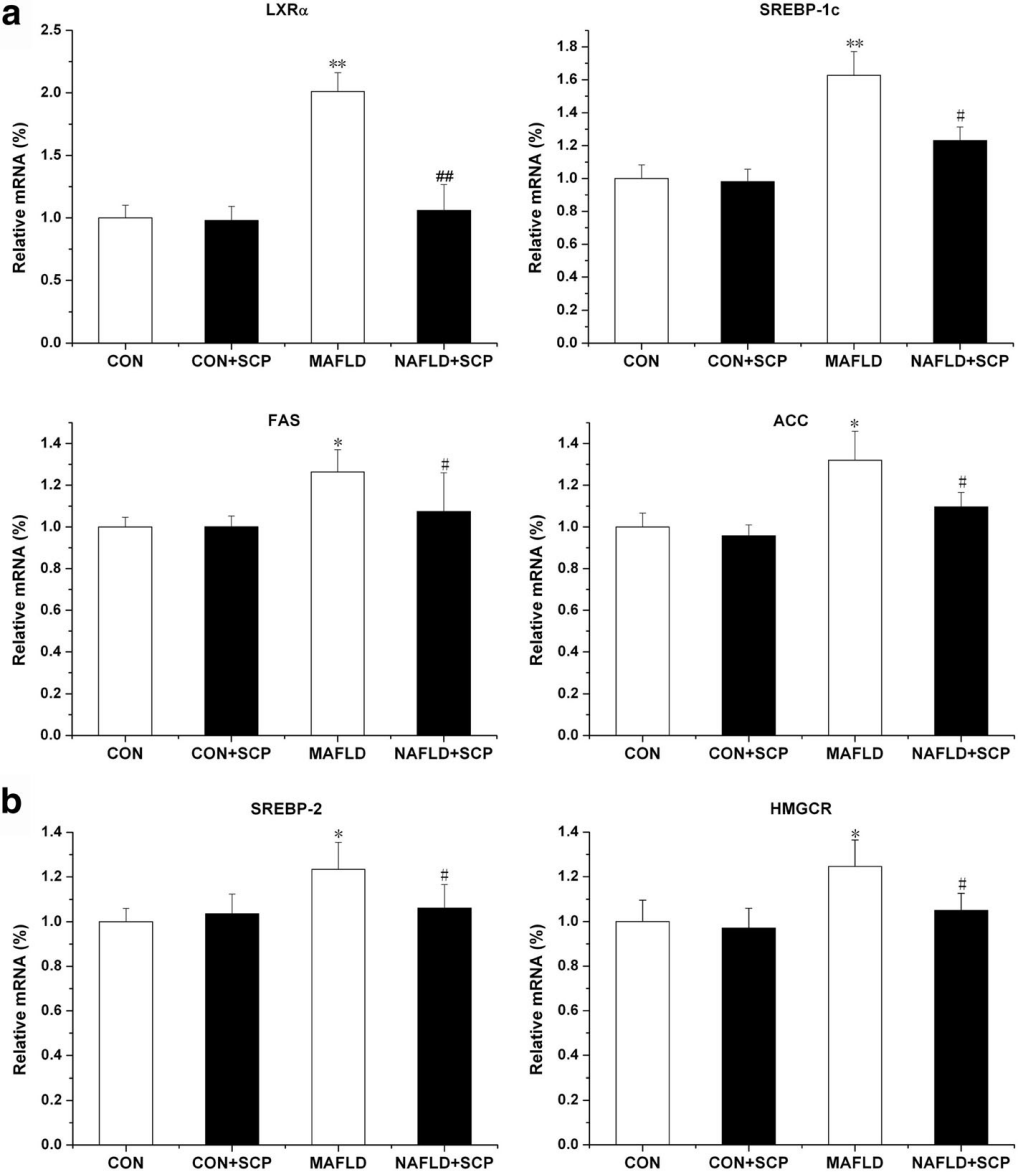
Effects of SCP on the mRNA expressions of lipogenic and cholesterol-related synthetic enzymes. **a** mRNA expressions of lipogenic enzymes (LXRα, SREBP-1c, FAS, and ACC) in the liver of mice were suppressed by SCP (100 mg/kg/day); **b** mRNA expressions of cholesterol-related synthetic enzymes (SREBP-2 and HMGCR) in the liver was suppressed by SCP-100 mg/kg/day. All data are expressed as mean (± SD) (*n* = 10) from three independent experiments; Between-group differences were assessed using one-way ANOVA with post hoc test. ^﻿﻿﻿**^ *P *< 0.01, ﻿﻿^*^ *P* < 0.05 *vs.* CON and CON + SCP groups; ^ ﻿##^ *P *< 0.01, ^#^ *P* < 0.05 *vs.* model group. *SCP, Schisandra polysaccharide; NAFLD, non-alcoholic fatty liver disease; SD, standard deviation; ANOVA, analysis of variance*

**Fig. 4:**
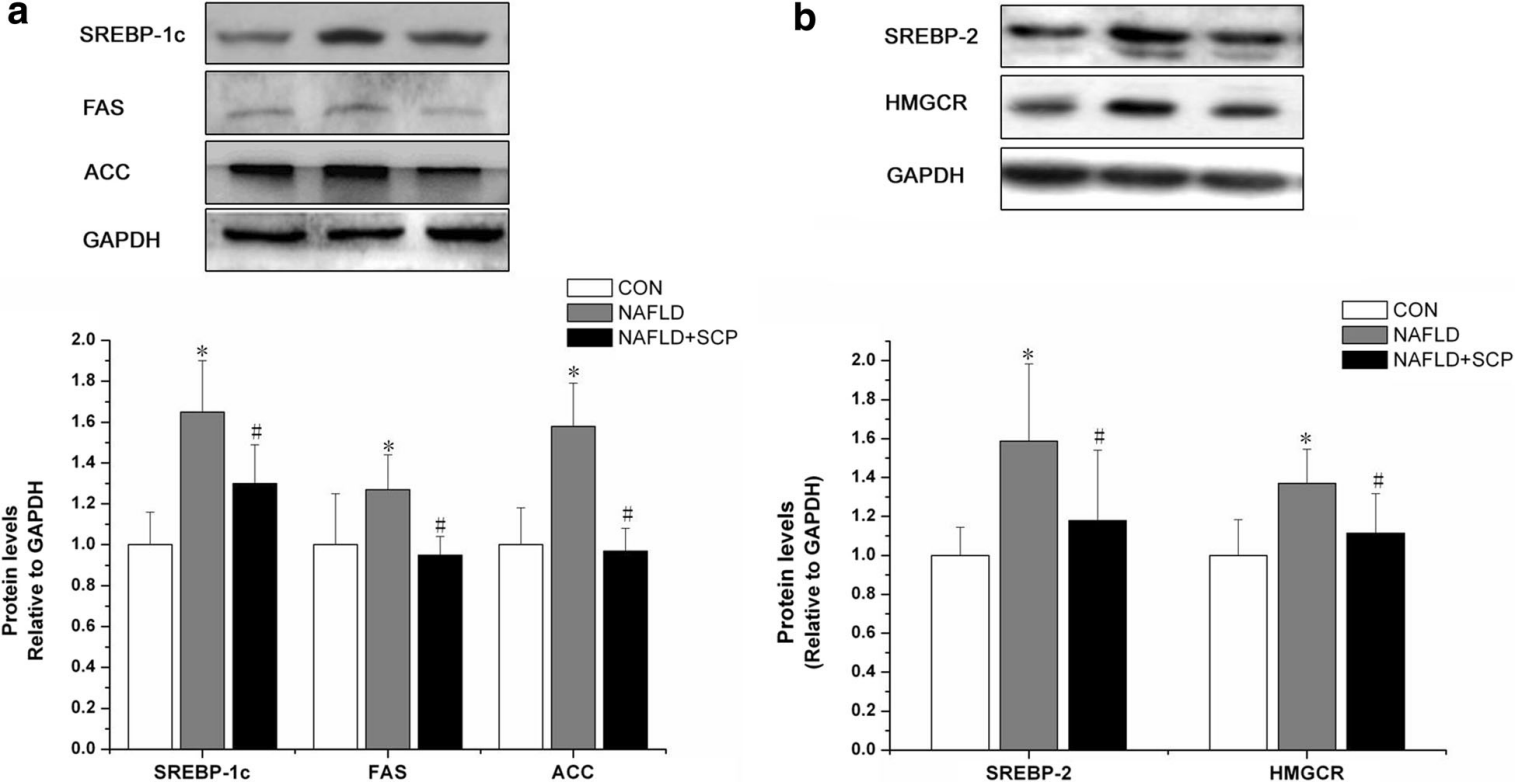
Effects of SCP on protein expression of lipogenic and cholesterol-related synthetic enzymes. **a** Protein expression of lipogenic enzymes (SREBP-1c, FAS, and ACC) in the liver of mice was suppressed by SCP-100 mg/kg/day; **b** The protein expression of cholesterol-related synthetic enzymes (SREBP-2 and HMGCR) in the liver of mice was suppressed by SCP-100 mg/kg/day. The protein levels are expressed relative to GAPDH. All data are expressed as mean (± SD) (*n* = 10) of three independent experiments; Between-group differences were assessed using one-way ANOVA with post hoc test. ^*^ *P* < 0.05 *vs.* CON and CON + SCP groups; ^#^ *P* < 0.05 *vs.* model group. *SCP, Schisandra polysaccharide; SD, standard deviation; ANOVA, analysis of variance*

As shown in Figs. 3 and 4, mRNA and protein expression levels of hepatic lipogenesis genes such as SREBP-1c, FAS, and ACC, were found to be increased in the NAFLD mice. Treatment with SCP inhibited the mRNA and protein expression levels. Furthermore, mRNA expression levels of LXRα, which can activate the transcription factor, SREBP-1c, were significantly decreased in the NAFLD + SCP group. In addition, the expression levels of SREBP-2 and HMGCR, which are key regulatory factors for cholesterol synthesis in the liver, were significantly increased in the NAFLD mice. The expression of SREBP-2 and HMGCR was significantly decreased in the NAFLD + SCP group. These data strongly suggest improved hepatic lipid metabolism after treatment with SCP.

## Discussion

*Schisandra* is the dried ripe fruit of *Schisandra chinensis* (Turcz.) Baill, a *Schisandraceae* plant, initially recorded in Shen Nong’s Materia Medica and classified as a top grade. It has long been known as the “king of protecting liver” on account of its hepatoprotective effects. Polysaccharides are the main active constituent of *Schisandra* and its content accounts for about 10%.

Effects of a refined SCP were observed in mice with NAFLD. The results showed that SCP could significantly reduce the liver index, decrease the serum levels of aminotransferases, triglycerides, cholesterol and low-density lipoprotein cholesterol, increase the serum level of high-density lipoprotein cholesterol, lower the content of triglycerides and cholesterol in the liver tissue, and ameliorate lipid accumulation and fatty degeneration of hepatocytes in NAFLD mice.

Pathogenesis of NAFLD is complex; the “two-hit hypothesis” suggests that the excessive accumulation of lipids induced by disordered lipid metabolic in liver cells is a prerequisite for the development of NAFLD [14]. ACC and FAS, the key enzymes for the synthesis of fatty acids in the liver, are regulated by nuclear transcription factor SREBP-1c [15]. Studies have shown that the hepatic expression of SREBP-1c and its target lipid synthesis regulation genes, FAS and ACC, increase several times both in patients with NAFLD and in animal models such as transgenic mice, ob/ob mice (leptin deficiency results in an insulin resistance and hyperinsulinemia), and high-fat diet-fed mice and rats, which leads to deposition of a large amount of TG [16–20].

Gene expression of SREBP-1c is regulated by LXRα, a nuclear receptor; activation of LXRα increases the expression of SREBP-1c, FAS, and ACC, which results in increased deposition of TGs in the liver [21]. In animal models and patients with NAFLD, the expression of LXRα/SREBP-1c significantly increases and shows strong positive correlation [22]. Consistent with these reports, our results showed that hepatic expression of LXRα, and SREBP-1 were significantly increased in the liver of NAFLD mice, and that SCP down-regulated the expression of LXRα and SREBP-1c.

Furthermore, SCP could significantly reduce the expression of SREBP-2 and HMGCR in NAFLD mice. In recent years, the role of abnormal cholesterol metabolism in the pathogenesis of NAFLD has attracted wide attention [23]. In 2007, Puri et al. [24] reported a significant increase in free cholesterol in the liver of patients with NAFLD. Caballero et al. [25] verified that in patients with NAFLD, not only free cholesterol but also SREBP-2 levels increased significantly. Furthermore, accumulated cholesterol is oxidized to cholesterol oxides, which further aggravates fat accumulation in liver cells via activation of LXRα/SREBP-1c pathway and upregulation of fatty acid and triglyceride synthesis [26, 27]. Our results suggest that SCP may alleviate the toxicity due to cholesterol accumulation in the mouse model of NAFLD induced by high-fat diet.

In China, *Schisandra* is listed as a raw material for nutraceuticals. Studies have shown that functional food and nutraceuticals have the potential to become the future of primary prevention and treatment of dyslipidaemia in particular, and cardiovascular disease prevention, in general [28]. The hepatoprotective and hypolipidemic effect of *Schisandra* makes it a promising candidate for development as a possible nutraceutical.

## Conclusion

In this study, SCP, an active component of *Schisandra*, improved liver function and alleviated fatty degeneration in the liver in NAFLD mice, an effect which appears to be mediated via down-regulation of LXRα/SREBP-1c/FAS/ACC and SREBP-2/HMGCR signaling pathways in the liver. These results provide a theoretical basis for further development and application of *Schisandra*.

## Abbreviations

ACC: Acetyl-CoA carboxylase
ALT: Alanine aminotransferase
AST: Aspartate aminotransferase
FAS: Fatty acid synthase
HDL-C: High-density lipoprotein cholesterol
HMGCR: 3-hydroxy-3-methylglutaryl coenzyme A reductase.
LDL-C: Low-density lipoprotein cholesterol
LXRα: Liver X receptor α
NAFLD: Non-alcoholic fatty liver disease
SCP: *Schisandra* polysaccharides
SREBPs: Sterol regulatory element-binding proteins
TC: Total cholesterol
TG: Triglyceride
